# Overcoming barriers in the implementation of stroke care rehabilitation in a public hospital in Costa Rica

**DOI:** 10.3389/fstro.2024.1366957

**Published:** 2024-03-22

**Authors:** Beatriz Coto-Solano

**Affiliations:** Rehabilitation Service, Hospital Rafael Ángel Calderón Guardia, Caja Costarricense de Seguro Social, San José, Costa Rica

**Keywords:** stroke, stroke rehabilitation, low and middle-income countries, developing countries, Costa Rica

## Abstract

Stroke is a major public health concern in developing countries, where the burden of the disease is high and resources for care are often limited. While progress has been made in improving stroke care, many barriers still exist in providing adequate rehabilitation care for stroke survivors. In this paper we study the case of Costa Rica and how stroke care has been addressed in recent years. It is important to consider the particularities of Costa Rica when working on stroke rehabilitation. The existence of a socialized healthcare system, along with the consolidation of acute stroke management protocols, allows for the adequate management of the early stages. In addition to this, families play a key role in rehabilitation, particularly for a country where there is a lack of medium stay and long-stay rehabilitation centers. Therefore, providing training and education for families is essential in stroke case management. Looking toward the future, there is still a pending need to generate homogeneous stroke rehabilitation protocols throughout the national healthcare system, to ensure equitable access to health care, and to consolidate multidisciplinary groups. At the same time, the implementation of technologies is urgent, particularly considering their potential to help reduce waiting lists. Another goal is enhancing coordination with other state entities and NGOs to advance community, labor or educational reintegration of stroke patients.

## Introduction

Pabón-Páramo (2020) analyzes the burden of stroke in Costa Rica from 2009 to 2019 through the data from the Institute for Health Metrics and Evaluation. A rising trend in mortality, incidence, prevalence of strokes, as well as disability-adjusted life years (DALYs) was observed. The crude incidence rate of strokes in 2009 was 70.6 new cases per 10,0000 inhabitants, rising to 88.6 in 2019. As for DALYs, 2009 had a crude rate of 457.8 per 10,0000 inhabitants – a total of 19,910 DALYs – while in 2019 the crude rate increased to 610.4 DALYs (total of 28,789 DALYs, an increase of 44%).

Evans et al. (2016) describe how the mortality adjusted rates for cardiovascular diseases in Costa Rica from 1970 to 2009 for ages 35-74 decreased by 58% (from 55 to 23). Despite the great success this number implies, as mortality diminishes, there is an increasing number of survivors with rehabilitation needs, which demands strategies to reduce morbidity and disability. Hospital Rafael Ángel Calderón Guardia (HRCG) receives an average of approximately 700–800 new stroke cases every year (Barboza, 2024). There is no available data on how many of the individuals affected by a stroke require rehabilitation and how many of these are actually able to access it.

Regarding acute settings, hospital-acquired complications have been documented in as many as 17% of the patients, including bronchopneumonia, urinary tract infections, dysphagia requiring PEG tube, and pressure ulcers, among others (Garro-Zuñiga et al., 2018; Serrano-Castro et al., 2023). Early rehabilitation is key in the prevention and rapid detection of these complications, as this allows for both early detection of deficits and work on recovery (Francisco et al., 2021).

There have been recent improvements in the management of acute stroke in Costa Rica, particularly with the introduction of the National Stroke Policy in 2018 that allowed for the homogenization of acute stroke medical management across the country and the enforcement of stroke units. However, there are still important unmet needs in terms of post-stroke rehabilitation and its introduction as an important part of the stroke unit. The national policy covers acute cases, but it does not incorporate measures for the rehabilitation process, which leads to late care of stroke patients at the expense of the critical recovery period. In addition to this, there is little integration within the neurology services of the rehabilitation staff, thereby reducing the possibility of a multidisciplinary approach.

At the same time, there is a lack of homogenization of protocols across different centers. Approaches are highly focused on motor deficits and there is little intervention in self-care, swallowing, language, cognitive and behavioral disorders, among others. There are clear geographical and architectural barriers, and existing stroke rehabilitation spaces are scarce, small, poorly equipped, and oversubscribed.

## Discussion

There have been significant efforts in the improvement of the stroke care in Latin America, and associations such as the Ibero-American Stroke Organization (SIECV), the World Stroke Organization (WSO) and the American Stroke Association (ASA) have become key stakeholders in this effort. In 2018, the Latin American Ministerial Meeting took place in Gramado, Brasil, creating a set of commitments -The Gramado Declaration- to be fulfilled to fight stroke in Latin America and provide better care for all. It included 16 recommendations around prevention of stroke, policy making, structuring of stroke centers, research and post stroke care rehabilitation (Ouriques Martins et al., 2019).

Ouriques Martins et al. (2019) reviews the 2-year advances of the Latin American Alliance for Stroke through a survey based on the Declaration of Gramado items. Regarding Costa Rica, a National Stroke Policy was implemented during this period, 5 stroke units were implemented, including 4 public and 1 private. Thrombectomy became available in 2 centers and access to rehabilitation post stroke was partially achieved, with both in and out- patient rehabilitation in place. There are also new stroke centers and stroke units. The integration of a network for continuous care of patients with stroke or stroke risk factors (that encompasses all levels of healthcare, creating a line of care) was not achieved, and there was no increase in research on stroke, based on the priorities and realities of the country.

In 2009, the Costa Rican Stroke Registry Program was created (Torrealba-Acosta et al., 2018). It has allowed for a few studies on epidemiological profiles and risk factors, as well as a PM&R thesis on functional outcome. Despite the steps forward reached in the acute care of stroke, as we dive deeper into the realities of stroke rehabilitation care, multiple barriers arise.

To further understand the rehabilitation care in Costa Rica it is important to understand where it stands within the national healthcare system. Costa Rica delivers healthcare through a national, public, socialized and universal institution named Caja Costarricense de Seguro Social (CCSS). It provides care to over 90% of the country's population through a geographically allocated, ascending complexity system that goes from primary health care to specialized centers. Four hospitals are classified as national; these are usually in charge of the more complex acute stroke cases. There are also seven regional hospitals and 13 peripheral hospitals which also provide care to acute stroke patients. Sometimes cases from the national centers are also referred to regional or peripheral hospitals for continuous care.

Regarding the rehabilitation system, there is one National Rehabilitation Center with 22 in-patient stroke beds and a National Geriatric and Gerontology Hospital with 35 in-patient hospital beds. The four national hospitals hold out-patient rehabilitation services, while seven regional hospitals, six peripheral hospitals and two clinics also offer these services.

Each center provides rehabilitation as its resources allow. As shown in Table 1, only five centers provide language therapy while only four centers provide occupational rehabilitation. Neuropsychological assessments and treatment are only provided in three centers. There is an insufficient amount of health providers and only one national PM&R residency program that is unable to replenish the physicians that retire or leave the public health system: There is an average of two retirements and 1 resignation per year, vs. four new PM&R physicians graduated per year. There are no specific protocols regarding the number of sessions patients receive and care is mostly based on a small number of sessions to train the patient and the caregiver.

**Table 1:** Stroke rehabilitation human resource availability in the Costa Rican public health system network.

**Center**	**PM&R**	**Physical therapy**	**Occupational therapy**	**Language therapy**	**Neuropsychology assessment and treatment**	**Social workers**
National rehabilitation center	x	x	x	x	x	x
National geriatric and gerontology hospital	x	x	x	x	x	x
H. Calderón Guardia	x	x	x	x		x
H. San Juan de Dios	x	x		x	x	x
H. México	x	x		x		x
H. Max Peralta	x	x				x
H. Heredia	x	x	x			x
H. Alajuela	x	x				x
H. Limón	x	x				x
H. Escalante Pradilla	x	x				x
H. Grecia	x	x				x
H. San Ramón	^*^	x				x
H. Liberia	x	x				x
H. Nicoya	x	x				x
H. San Carlos	x	x				x
H. Puntarenas	x	x				x
H. Guápiles	x	x				x
H. Turrialba		x				x
Clínica Carlos Durán		x				x
Clínica Coronado	x	x				x
Clínica Clorito Picado	x	x				x

X stands for the presence of this specific service on the medical center site.

Rehabilitation tends to be more focused on mobility deficits, leaving aside other issues such as language, cognition, self-care and behavior, undermining the possibility of an improvement in the quality of life and ignoring the future complications and burden of care that consequently might arise. Regarding access to assistive products, as surveyed at the outpatient rehabilitation services through the public system using the WHO rATA tool (Coto-Solano, 2022), 47% of users consider having an unmet need (whether it was the need for a new product or a change in the one they have) with the main barrier being unaffordability within a system that mainly focuses on providing mobility aids, leaving aside visual, cognition, selfcare and language aids.

The system also faces an overwhelming number of users in need of treatment, who must quickly receive their rehabilitation to get the most advantage of the early recovery period. Nevertheless, the waiting lists are beyond what the system can handle, with wait times for a PM&R consultation of about 2 months and waiting time for therapy of ~3 months.

There are several ongoing strategies to solve these unmet needs. At HRCG we received optional consultations from the stroke unit to evaluate patients. After this, a sole visit was performed, and follow-ups were not scheduled. Reference was sent by the treating physician as the patient was sent home so an outpatient appointment might be assigned. When the appointment came, the therapy was prescribed. Despite all of this, a few months could pass before the patient received the first outpatient therapy. No formal training was given to the family to cope with the patient's needs at home. Recently, our group has sought to increase the links between rehabilitation services and stroke diagnostic services. The multidisciplinary approach is not only implemented during the hospital stage, but it is considered thoroughly during the continuum of care. Early stroke rehabilitation care has been implemented and currently, whenever patients are admitted with a diagnosis of stroke, they are referred to rehabilitation on the same day. In this way, it is possible for a Physical Medicine and Rehabilitation physician (PM&R) to perform a first early assessment, including chart reviews, family member interviews, and information gathering. After this, neurological and functional assessments are completed, and the goal planning is set accordingly. Interventions by the rehabilitation team, including physical, occupational and speech therapy, are provided through in-patient time, and coordination with a social worker is done to facilitate discharge. Though desirable, at this point we do not yet have a neuropsychologist in the team to complement the assessment and treatment. To date, it is not possible to address all the deficits in the treatment of stroke patients, but there is an active effort to improve it. This very early effort focuses mostly on the family members' training on skills such as transfers, safe eating, bathing, prevention of pressure ulcers, cognitive stimulation, basic therapeutic exercise, and basic home adaptations.

Other specialists are beginning to be incorporated into rehabilitation services, including those that deal with complications of stroke patients, such as specialists in psychiatry, geriatrics, urology, otorhinolaryngology, gastroenterology, dieticians, clinical nutrition and neurosurgery.

When a discharge is set, follow up rehabilitation is planned. This could be an in-patient period in the National Rehabilitation Center or the National Geriatrics Hospital, where a period of 2 months wait listing is expected; or the treatment could start in an outpatient setting, whether in the national, regional, peripheral hospital or clinic, with a wait listing period of 2 months as well. Follow up by a PM&R physician is also set. As waiting times can extend for both in and outpatient, patients return to their home to wait. Ideally, the family members and the patients will follow all the recommendations given by the rehabilitation team when released from the hospital to avoid any complications and they will continue to do the exercises that they were taught beforehand. This will allow for the patient to continue improving at home. The training of the patient and the family before leaving the hospital is one of the key features of the rehabilitation program, given the fundamental role they play in the patient's recovery.

The out-patient period can include a small number of sessions and thereby prove insufficient for the therapy. Different efforts have been made to translate technology developed by universities within Costa Rica and outside the country in order to ameliorate the waiting list issue and the limited amount of therapy sessions provided. Unfortunately, complex barriers are faced (Coto-Solano, 2020) including a very inflexible and bureaucratic health system that complicates the partnering not only with the private sector but with the very same public sector it is part of. There is also an important lack of research experience within the Costa Rican rehabilitation sector, lacking not only formal research training but also proper protocols to accompany clinical care and growing areas such as the local device production.

After the out-patient period a lack of connection to the patient's community is evident. Different stakeholders such as municipalities, universities, sport committees have programs that are not coordinated among them, but certainly are an interesting ground to explore in order to create a continuum of care from the acute setting to the community setting. These stakeholders are also a basic tool in secondary stroke prevention, including continuous physical activity, mental health, smoking cessation and weight control. This might also prove to be a useful tool in a setting with important geographical barriers such as Costa Rica, where long distances might need to be traveled to access rehabilitation care. This will be the next strategy that our team will explore.

There is also an urgent need to not only advocate for multidisciplinary groups – for which there is vast evidence of better outcomes – but to make them a reality. Not only is this a quality-of-life matter but even a cost-wise one, since enhancing the probabilities of functional recovery of an individual to come back to work/school/life and reinsert themselves into their community must be the final goal. Unfortunately, throughout the territory most rehabilitation is focused on motor deficits, leaving behind any other functional issue. This is clearly reflected in the composition of the rehabilitation services (Table 1). The data we have generated has helped inform decision-makers on the need to strengthen our services with personnel that go beyond physical therapists, including occupational and speech therapists, psychologists, and social workers, among others. Introductory rehabilitation medicine courses to undergraduate medicine students are also taught and parallel measures are being taken to guarantee the training of a larger number of residents to properly meet the need of PM&R physicians.

Another important step will be the creation of a national stroke rehabilitation protocol, as well as policies like the ones created for stroke acute care, where common protocols might be developed with proper key performance and quality of care indicators to standardize rehabilitation stroke care and allow for resources to be properly allocated, facilitating the continuum of care previously discussed and supporting the secondary stroke prevention. Discussions are already ongoing with colleagues from the acute stroke care to develop these protocols. There is also a need for creating appropriate structural spaces for the rehabilitation care, which are now scarce.

Another key aspect that is lacking is the inclusion of the user perspective in the continuous improvement of stroke rehabilitation services. The only national survey regarding the delivery of rehabilitation services in Costa Rica reviewed access and satisfaction regarding assistive products (Coto-Solano, 2022), and at the time of writing there is not stroke patient association that could support stroke initiatives or exert political pressure to ensure their existence.

Finally, following the footsteps of stroke acute care, it is important to create stroke rehabilitation networks throughout the region, creating the possibility of sharing experiences, knowledge and solutions within our contexts and realities.

## Conclusions

Despite the multiple barriers faced by stroke rehabilitation worldwide and particularly in low- and middle-income countries such as Costa Rica, efforts must be sustained for the implementation of multidisciplinary rehabilitation interventions with updated policies and protocols regarding stroke rehabilitation adapted to the country's reality.

## Data availability statement

The original contributions presented in the study are included in the article/supplementary material, further inquiries can be directed to the corresponding author.

## Author contributions

BC-S: Writing – original draft, Writing – review & editing.
